# Illness recognition and care-seeking for maternal and newborn complications in rural eastern Uganda

**DOI:** 10.1186/s41043-017-0125-x

**Published:** 2017-12-21

**Authors:** Monica Okuga, Peter Waiswa, Rogers Mandu, Juddy Wachira, Claudia Hanson, Fatuma Manzi

**Affiliations:** 1 Department of Health Policy, Planning and Management, Makerere University School of Public Health, Kampala, Uganda; 2 Center of Excellence for Maternal, Newborn and Child Health, Makerere University, Kampala, Uganda; 3 Department of Public Health Sciences, Karolinska Institutet, Stockholm, Sweden; 4 School of Medicine/AMPATH, Moi University, Nairobi, Kenya; 5 London School of Hygiene and Tropical Medicine, London, UK; 6 Ifakara Health Institute, Dar es Salaam, Tanzania

## Abstract

**Background:**

To enhance understanding of the roles of community-based initiatives in poor rural societies, we describe and explore illness recognition, decision-making, and appropriate care-seeking for mothers and newborn illnesses in two districts in eastern Uganda where in one implementation district, a facility and community quality improvement approach was implemented.

**Methods:**

This was a cross-sectional study using qualitative methods. We conducted 48 event narratives: eight maternal and newborn deaths and 16 maternal and newborn illnesses. Additionally, we conducted six FGDs with women’s saving groups and community leaders. Qualitative data were analyzed thematically using Atlas.ti software.

**Results:**

Women and caretakers reported that community initiatives including the presence of community health workers and women’s saving groups helped in enhancing illness recognition, decision-making, and care-seeking for maternal and newborn complications. Newborn illness seemed to be less well understood, and formal care was often delayed. Care-seeking was complicated by accessing several stations from primary to secondary care, and often, the hospital was reached too late.

**Conclusions:**

Our qualitative study suggests that community approaches may play a role in illness recognition, decision-making, and care-seeking for maternal and newborn illness. The role of primary facilities in providing care for maternal and newborn emergencies might need to be reviewed.

## Background

With the adoption of the Sustainable Development Goals, new and ambitious targets have been set for reducing maternal and neonatal deaths globally: by 2030, the global maternal mortality rate (MMR) should not be higher than 70 per 100,000 live births and neonatal mortality rate (NMR) not more than 12 per 1000 live births [1]. In Uganda, the MMR is 368 deaths per 100,000 live births, while the NMR is 27 per 1000 live births [2]. Achieving these global targets will require that maternal and newborn illness or complication is recognized early, a decision to seek care is appropriately made followed by timely care-seeking, and when facilities are reached, quality care is available and provided. However, as described by Thaddeus and Maine [3] for maternal deaths and Waiswa et al. [4] for newborn deaths, care-seeking delays are common and result in severe morbidity and mortality. Understanding how illness recognition, timely decision-making, and appropriate care-seeking can be achieved effectively and sustainably, and at scale is important for building efficacious national programs.

The primary delivery of health care and evidence-based interventions for maternal and newborn health is mandated in Uganda to administrative districts as per decentralization policy [5]. Particular implementation focus is on rural districts, as they often have the highest mortality rates [6]. However, there is limited evidence in the literature on how districts best operationalize access to care including illness recognition, timely decision-making and appropriate care-seeking.

In Mayuge district of eastern Uganda, a district-wide maternal and newborn intervention study, the Expanded Quality Management Using Information Power (EQUIP) project [7] implemented a community quality improvement approach based on national community health worker (CHW) strategy. EQUIP aimed to improve maternal and newborn care. The neighboring Namaingo district served as a comparison district for this plausibility trial which is explained elsewhere in detail [7]. In short, the EQUIP quality improvement approach (2010–2014) was based on the collaborative model of improvement [8] and linked communities, health facilities, and district health management teams. At the community level, quality improvement teams (QIT) were formed by CHW at the village level to work on local problems which hamper the implementation of WHO essential interventions [9]. Teams were encouraged to analyze locally identified problems––including delayed care-seeking for maternal and newborn care––and to generate possible solutions. The solutions, called “change ideas” were then tested within the community, and their effects were assessed using locally generated data on maternal and newborn indicators. Examples of change ideas included visiting of pregnant women to counsel women, identify maternal and newborn danger signs and refer if necessary, using an approach and materials developed as part of the Uganda Newborn Study (UNEST) and the establishment of community savings funds (women’s savings groups) [7, 10].

In view that EQUIP provided a novel approach of collaborative quality improvement using CHWs to improve illness recognition and care-seeking, we aimed to understand how maternal and newborn illness recognition, timely decision-making, and appropriate care-seeking were enhanced. We did not set out to explore perceived quality of maternal and newborn care, although this inadvertently emerged during the interviews.

## Methods

### Study design, area, and intervention

This was a qualitative cross-sectional study conducted in the EQUIP intervention (Mayuge) and comparison (Namaingo) districts in eastern Uganda. Both are typical rural districts. Mayuge has a population of 460,000 while Namaingo has approximately 233,000 people [11]. Both districts lie on the Northern shores of Lake Victoria. This region has a fertility rate of 6.8, greater than the national average of 5.9 [12]. Namaingo has 22 health centers and no hospital, and Mayuge district has 40 health centers and one private-not-for-profit hospital that charge fees for services.

### Study population and sampling

We selected 48 mothers; 24 each from Mayuge and Namaingo districts (Table 1) aged 18–49 years, and caretakers of women and babies (spouses, neighbors, friends, siblings) who fell ill or died from four purposefully selected parishes (two rural and two peri-urban). In each selected parish, with the help of the CHWS, we identified one maternal death, two cases of excessive bleeding, two newborn illnesses, and one newborn death. The selection criteria for the cases were that they must have been home when the illness was recognized. Cases arising while in hospital were excluded. Case definitions are further described in the protocol paper in this supplement [13]. Six focus group discussions (FGDs) were conducted in both the intervention and comparison districts (Table 1) to gain perspectives on illness recognition and care-seeking with community leaders and women’s savings groups. Women’s savings groups are groups of 50–70 women within the community who make a monthly contribution of $0.30 that can be borrowed to facilitate health care-seeking. These savings groups were part of the change ideas initiated and used in the Mayuge district only. Potential cases for event narratives were identified with the help of CHWs in the selected parishes. In Mayuge district, the CHWs keep updated registers where they record all pregnant women, maternal deaths, newborn deaths, deliveries, and women who have had maternal complications such as post-partum hemorrhage, newborn illnesses, and prematures within their parishes. However, in Namaingo, even though the CHW structure is in place, it is not fully operational. Further screening of identified cases was conducted using a checklist to determine true cases. A screening checklist was used to screen cases and determine eligibility.

**Table 1:** Sample size of each category of respondents in each district

Case type	Mayuge (intervention)	Namaingo (control)	Total cases/interviews
Maternal			
Women who perceived to have had excessive bleeding after birth and survived (maternal illness)	8	8	16
Women who died within 42 days of birth regardless of cause (maternal death)	4	4	8
Newborn			
Women who perceived their newborn became ill before 28 days of life and survived (newborn illness)	8	8	16
Women whose newborn became ill and died within 28 days of life (newborn death)	4	4	8
FGDs with community leaders	2	2	4
FGDs with women’s groups	2		2
Total number of interviews	28	26	54

### Data collection

The interviews and FGDs were conducted from April to July 2015 by four trained Research Assistants (RAs): two data collectors and two note takers, chosen on the basis of prior experience with qualitative interviews. All the qualitative interviews were conducted in the local language, Lusoga.

The research team screened the previously identified households to locate cases of maternal excessive bleeding after delivery, maternal deaths, neonatal illness, and neonatal deaths using the inclusion criteria as described above in the study population. Willing participants were then asked to identify 2–3 individuals who were present and provided support during the maternal/newborn illness or death. In some cases, where respondents were available, the group interview was conducted immediately, while in other cases, an appointment had to be made. In most of the cases, the narratives were done in a single interview with all the care givers in the family. However, in some cases, for example, where the mother seemed uncomfortable speaking in the presence of other relatives, an additional separate interview was conducted with her.

For the FGDs, eight community leaders/members of the women’s groups were identified and selected as respondents per FGD with the help of the CHWs. Informed written consent was obtained from each respondent.

Quality assurance was maintained through a number of steps. Prior to the study, the RAs underwent a 4-day training on study objectives, methods, and ethical considerations. Qualitative data collection tools were translated into Lusoga and back translated to English by different people to ensure their accuracy. All discussion guides for narratives and FGDs were pretested in one of the parishes not included in the sampling frame. To ensure quality, audio recordings were checked against the transcripts. All translations were double checked. Daily field notes were also used for quality checks. Intermittently, the study manager supervised data collection in the field.

### Data analysis

Interviews were transcribed verbatim. The qualitative data analysts read through all transcripts and field notes from group interviews and FGDs multiple times to familiarize themselves with the data. Complete event narratives were written for each index case using data from the interviews. Content analysis methodology was used to code the transcripts using Atlas.ti software [14]. The responses were coded by case type. Any code with less than 100% inter-coder agreement was re-evaluated and dropped if there was no agreement. This analysis included coding within cases and comparing across cases and between intervention and comparison areas, identifying crisis recognition categories and broad themes. Key narrative domains and sub-domains include perceived signs and symptoms (what and by whom), care-giving at home (what, by whom, when in relation to signs, and symptoms), care-seeking decision-making (whether it occurred or not, involving whom, what were the considerations), care-seeking (from whom, in what sequence, barriers encountered), perceived cause of the illness, and contextual factors that may have influenced recognition of and response to the illness. To provide depth to and illustrate the model, we also coded narrative quality, noting the presence, content, and resolution of contradictory statements among informants in the group. The analysis model was in the form of a table with four domains that contained key themes and sub-themes which were explained chronologically by multiple factors to authenticate the phenomena.

### Ethics approval and consent to participate

This research was approved by the Makerere University School of Public Health Higher Degrees Research and Ethics Committee and Uganda National Council of Science and Technology. Permission was also obtained from the Mayuge and Namaingo District Health Offices. Written informed consent was obtained from all participants using a consent form translated to Lusoga for clear understanding. All data was treated with confidentiality and presented only in aggregate form or anonymized. Confidentiality was ensured throughout the study. Audio recordings of the illness narratives and focus group discussions were kept in a locked room and will be destroyed after 5 years in accordance with the IRB rules.

## Results

We conducted 48 event narratives (8 maternal deaths, 16 maternal illnesses, 8 newborn deaths, and 16 newborn illnesses) and 6 FGDs. There were two refusals (cases related to one newborn death and one maternal death) for the event narratives, but these were replaced. Focal persons (key caretakers) in the maternal illness and death cases were mostly the husbands and mothers-in-law. In some cases, sisters and friends were involved. All the respondents for the newborn event narratives were female, and the majority were the biological mothers. Demographic characteristics of the focal person are presented in Table 2.

**Table 2:** Demographics of maternal respondents

Variable	Category			
	Maternal death (number/%age) *N* = 8	Maternal illness (number/percentage) *N* = 16	Respondents for newborn illnesses (number/percent) *N* = 16	Respondents for newborn death (number/%) *N* = 8
Age, 0–18 years	0 (0)	2 (12.5)	0 (0)	1 (12.5)
19–24 years	2 (25)	5 (31.25)	10 (62.5)	5 (62.5)
> 25 years	6 (75)	9 (56.25)	6 (37.5)	2 (25)
Parity 1	3 (37.5)	1 (6.25)	3 (18.75)	2 (25)
2	1 (12.5)	6 (37.5)	4 (25)	4 (50)
3 and above	5 (62.5)	9 (56.25)	9 (56.25)	2 (25)
Marital status				
Married	8 (100)	14(87.5)	13 (81.25)	
Single/widowed/separated	0 (0)	2 (12.5)	3 (18.75)	

We grouped findings in three domains: (1) illness recognition and care-seeking for maternal and newborn cases, (2) role of community initiatives in illness recognition and care-seeking, and (3) patient experiences with care.

### Maternal and newborn illness recognition and care-seeking

We noted differences in terms of illness recognition, decision-making, and care-seeking between mothers and newborns (Table 3).

**Table 3:** Differences between maternal and newborn cases

Domain	Maternal cases	Newborn cases	Intervention area	Comparison area
Illness recognition	Illnesses fairly quickly recognized	Delay in recognition of illness––symptoms	Illness seems to be more quickly recognized Interviews reveal better knowledge of danger signs Knowledge attained from the VHTs and Health workers Minimal influence of culture	Larger delay in Illness recognition Less knowledge of danger signs Culture plays big role in delay in recognition
	Cause of illness and death mainly reported as biological	Cause of illness and death unknown and mainly reported as God’s will. Other causes reported are witchcraft		
Decision makers	The focal woman, husband, and mother in law are key decision makers	The mother and mother in law are key decision makers Husband only contacted when a referral is required or the illness is complicated		
Care-seeking	Care sought mainly from formal health care providers Although informal in a few cases	First care-seeking point is home. Home remedies tried first before others	Care sought from multiple points up to 4 steps Most cases sought care from government health facilities and private clinics	Care sought from 1 to 2 points Care sought mainly from government facilities and informal providers
	Care-seeking relatively fast	Delay in care-seeking		
Community initiatives influence			Women report about the helpfulness of VSLAs/women’s groups to facilitate care-seeking	No women’s savings groups

### Maternal and newborn illness recognition

In both the intervention and comparison areas, recognition of maternal complications and danger signs was common. Maternal illness symptoms of excessive bleeding were generally quickly recognized as heavy bleeding with clots, bright red blood gushing out, having clothes and mattresses drenched with blood and frequently changing sanitary pads.

*“…..I was bleeding so much that whenever i put a pad, it would immediately get soaked with blood and I would have to change. I then knew that there was a problem” (maternal illness, 21-year-old mother, intervention)*.

Other maternal illness symptoms recognized included loss of consciousness, headaches, swelling of the body during pregnancy, dizziness, being bed ridden, and inability to do household chores. All the maternal illness cases in the intervention area were quickly recognized as opposed to four of the eight cases in the comparison area. Unlike post-partum bleeding, newborn illnesses which are a much broader category of conditions were not easily nor quickly recognized. The newborn illness signs that were commonly recognized included baby crying excessively, fever, failure to breast feed, difficulty in breathing, and diarrhea. Yellowing of the skin was one of the signs less easily recognized. The respondents who have babies with jaundice had initially thought the babies were well until they worsened.

In both intervention and comparison areas, respondents were able to mention the biomedical causes of maternal illnesses and death such as excessive bleeding after delivery, high blood pressure in pregnancy, and anemia. For example, excessive bleeding was stated as the cause of a mother being anemic.

*“They told me the bleeding was too much and the patient was bleeding non-stop so to me I feel the problem was this excessive bleeding, because they tried to give her the injection which stops bleeding but the bleeding continued so that was caused the woman to become anemic” [Maternal death, 31-year-old husband, intervention]*

On the contrary, the perceived causes of newborn illnesses and death were reported as unknown or associated with non-medical influences including “God’s will” and witchcraft. In very few cases, medical causes, for example prematurity, were cited.

*“I think it was traditional because there were some women who came to see me after I had delivered and I think they are the ones who caused the death of my baby as one is a well-known witch” [Newborn death, 20-year-old mother, comparison]*

### Decision makers

The key decision makers for maternal cases were the mothers-in-law, the focal woman herself, and her husband. In most cases, mothers-in-law were present at the time of the illness and influenced the decision-making process. Husbands were usually consulted even though they were sometimes not present in the home. In cases where the husband was away from home, the use of mobile phones was seen as critical in enhancing communication between husbands and the caretakers; however, this caused delays in making decisions.

*“Based on the phone calls I (the husband) was getting from the caretaker I knew the condition was not good at all and at every point of progression they were calling me but her condition was instead worsening. I asked them to proceed to the health facility while I also tried to find my way there to meet with them” [Maternal death, 40-year-old husband, intervention]*

Regarding newborn illnesses, the key decision makers were the mothers of the sick newborns and the mothers-in-law (husband’s mother). They determined where the baby would be taken for care and at what point of the illness. The role of the husbands was to provide finances for care-seeking.

*“Most times our husbands left responsibilities to us as mothers, there are few men who care that the baby is sick and they have to be taken to the health center but most times it is us the mothers who have to look for a way you get your family health care” [FGD, women’s group, intervention]*

### Care-seeking for maternal and newborn illnesses and deaths

**Fig. 1:**
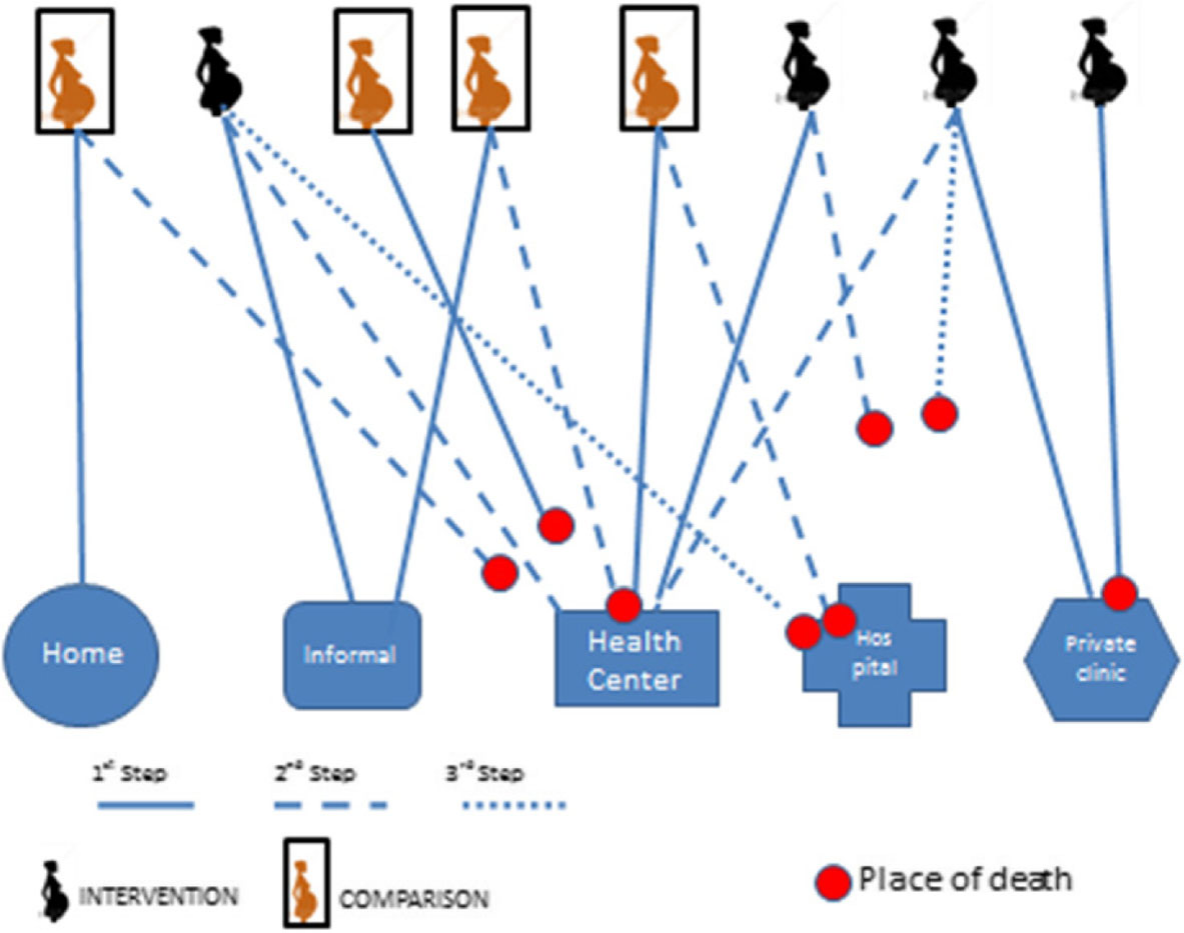
Care-seeking patterns for maternal deaths

Generally, care-seeking patterns were similar for maternal illnesses and deaths and likewise for newborn illnesses and deaths studied. Families reported that care was sought from several providers before death (Fig. 1). For example, mother 1 first tried to manage the complications at home and she died on the way to a health center. Mother 2 went first to seek care from an informal provider, then to a health center, and died then finally while seeking care in a hospital. The first point of care-seeking was the informal sector (1 of 8), the health center (1 of 8), and the private clinics (2 of 8) in the intervention district. In the comparison district, one mother sought care first at home, one in the informal sector, and one at the health center, and one died on the way to a health center. None of the mothers, either in the intervention or the comparison area, went straight to the hospital to seek care. The care-seeking steps involved for two mothers’ one step, four mothers’ two steps, and two mothers’ three steps. Four of the eight mothers died on the way to the health facility to seek care either during the first, second, or third steps. Two of the mothers died in a health facility, one in a hospital and one in a private clinic.

**Fig. 2:**
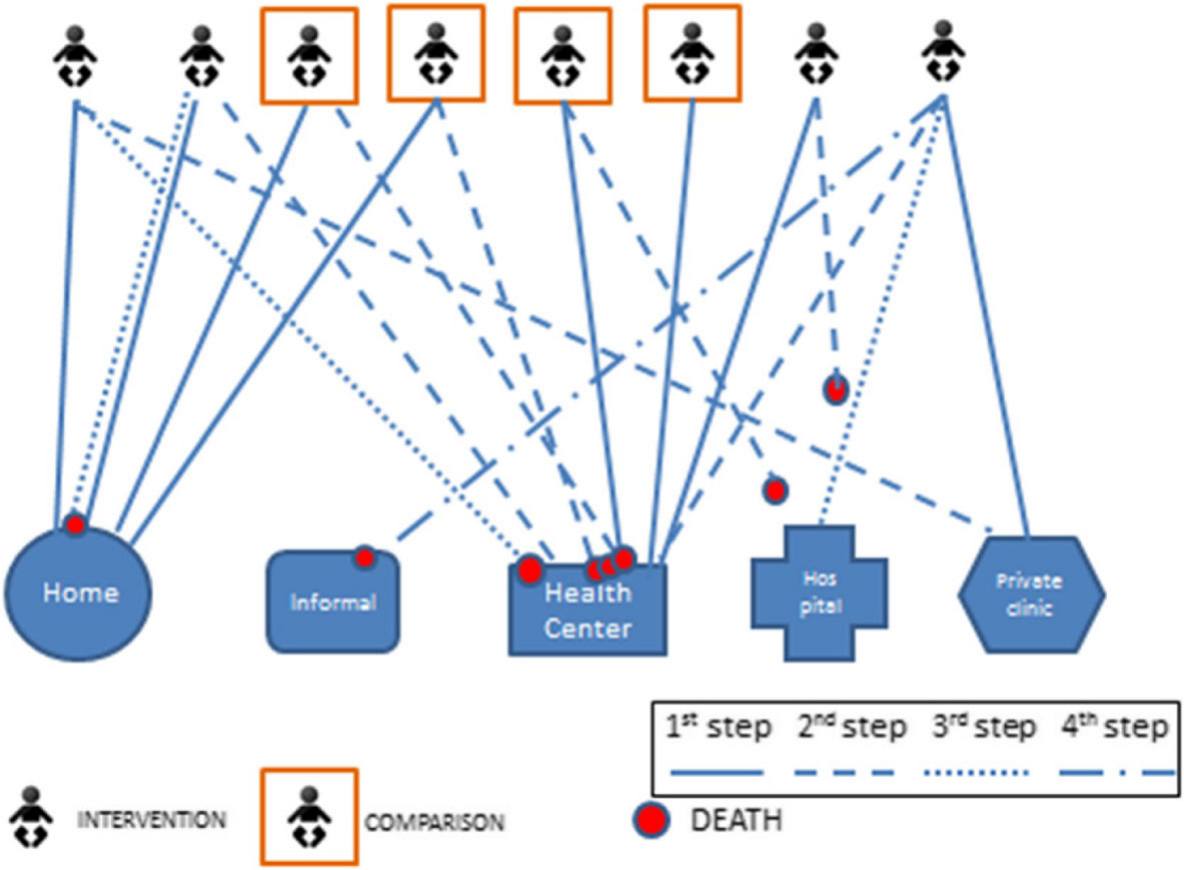
Care-seeking patterns for newborn illnesses

The first point of care was at home for four and at a health center for three of the eight newborn deaths (Fig. 2). Remedies used at home included taking baths, tepid sponging, and use of herbs among other home remedies. Four of the eight cases visited a health facility as the second step of care-seeking. Only one baby was brought to a hospital. Two babies died before reaching the hospital, while four babies died at the health center, one at home and one at an informal care provider’s (herbalists) practice. Care-seeking involved in one case, only one step; in four cases, two steps; in two cases, three steps; and in one case, four steps.

### Patient experiences with care

Quality of care was defined by the respondents often in terms of availability of drugs, presence of a health worker at all times, waiting time, health worker attitudes, user fees, and interpersonal relations. Provider-patient relationship was mentioned as one of the factors affecting utilization of services. Some respondents mentioned that the reason why they do not seek care from certain facilities is because the health workers are rude and unwelcoming. Notably, private health facilities were mentioned by nine of the respondents to have more client-focused care and friendly health workers as compared to public health facilities. Respondents mentioned that at the private facilities, they were likely to find health workers who were kind to them and gave them the necessary drugs required as opposed to public facilities.

*“I prefer going to the clinic because they always have drugs. At the government health facility, they always ask us to buy drugs from the pharmacy in town” [Maternal illness, 22-year-old mother, intervention]*

*“At the government facility, the nurses are always very rude and abuse you for no reason” [Maternal illness, 24-year-old mother, comparison]*

The waiting time was reported by most respondents as a determinant of which facility to go to. In some facilities, mothers had to queue for many hours before receiving services, especially in the public health facilities. This resulted in care-seeking from other places such as drug shops, private facilities, and in some cases, home remedies or no care sought at all.

*“You can line in a queue from morning to 1:00 pm, and that is when the health workers go for lunch, you sit and wait for them to come back at 3:00 pm and they write for you that there are no drugs, so most times I do not go there, the only time that I go there is when am pregnant.” [Newborn illness, 28-year-old mother, intervention]*

### Role of community initiatives in illness recognition and care-seeking for maternal and newborn illnesses

The role of the community initiatives in maternal and newborn illness recognition and care-seeking was more apparent in the intervention than the comparison area (Table 3). These community initiatives included the women’s saving groups initiated in the intervention area as a quality improvement change idea to help women access funds for health care.

The respondents from the 11 of the 12 event narratives (interviews) in the intervention area reported that they obtained knowledge about maternal and newborn danger signs mainly from formal health workers at the health facility, the CHW, or the Village Health Team (VHT) through village sensitization meetings and home visits. They reported further that CHWs used a visual aid to convey information on the danger signs, which was not the case in the comparison district.

*“I learned about danger signs in mothers and babies through the VHT who visited my home while I was pregnant. So when my baby started breathing badly, I knew I had to go to a health facility” [Newborn illness, intervention]*

Respondents from the case interviews and FGDs in the intervention area mentioned factors such as presence of village saving groups/women’s groups, CHW, and community support systems as influencers of care-seeking. The presence of village saving groups, especially the women’s saving groups, was reported as a chief facilitator of care-seeking in the intervention district. Saving groups were not present in the comparison district as this was one of the change ideas initiated by the EQUIP project at community level.

*“In our women savings groups, when a woman reaches a time to deliver or has any sickness or her child is sick, she goes and she is given money and after she refunds it and it is given to another.” [FGD, women’s group, intervention]*

CHWs were also mentioned as key influencers of care-seeking especially in the intervention area. All respondents in the intervention area and half of the respondents in the comparison area stated that usually when they experienced an illness, they would first consult with the CHW who would either refer them or give them the necessary medication. Consultation was either by phone or in person.

*“The VHTs/CHWs are very helpful. We always ask them when we have problems and they tell us what to do…either to go buy certain medicines or go to the health facility.” [FGD, community, intervention]*

In both the intervention and comparison areas, generally, community members like neighbors/friends also facilitated care-seeking by being caregivers, looking after the home or children when the sick mother sought care, or contributing food and money for care-seeking as well as for institutional delivery.

*“The people in the village helped and gave me money for the transport I used to go to the health facility to deliver and to come back home. When I came back people brought for me sugar and soap.” [Maternal illness, 38-year-old mother, comparison]*

## Discussion

In this study, we observed that illnesses such as post-partum hemorrhage which typically presents with clearly noticeable signs and symptoms were well recognized. In contrast, respondents reported greater difficulties in interpreting the more unspecific signs and symptoms of newborn illnesses. Care-seeking for both maternal and newborn death involved several steps and referrals. Often, a pattern from care-seeking at one or more facilities belonging to the primary level was described. In no case was the first point of care-seeking a hospital.

Illness recognition is a critical step to making a decision to seek care. From our study findings, newborn illness was not well understood and formal care often delayed. The challenge in recognition of newborn illnesses may have been due to lack of specific symptoms or even masking of symptoms. This finding was similar in other settings in Uganda [4, 15, 16] and in Tanzania [17]. It may also be that the symptoms are actually seen, but severity is not recognized. Mothers and families seemed to be able to recognize bleeding after birth (post-partum hemorrhage), lack of blood (anemia), fever (which they called malaria), and hypertension in pregnancy (which they identified with swelling of the feet and the face). Respondents from the intervention districts explained that CHWs facilitated recognition of illnesses through sensitization of communities on maternal and newborn danger signs and hence reducing delay one [15, 18]. These CHWs often advised and even referred patients to the formal facilities for care. Such community initiatives have been successfully implemented elsewhere for neonatal illnesses leading to an increase in care-seeking behaviors and improved newborn survival [16, 19].

In order for a mother to seek care, decision-making was influenced by both the male partner or by older women such as a mother-in-law. However, men had limited influence in care-seeking for newborn illness. This being a patriarchal society, men still dominate economic power and related decision-making [20]. Therefore, for most cases, the spouse/husband had to be contacted even when he was away from home. The mobile phones played a key role in communication with husbands who were away from home especially with regard to maternal illnesses. On the contrary, interviews for neonatal illnesses found that the mother of the sick newborn and her mother-in-law were the chief decision makers. This may have been due to the fact that informal care sought for the newborns has a less bearing on economic resources. In this setting, perhaps based on presumed experience, the mothers-in-law or older women seem to wield influence in terms of illness recognition and decision-making on whether to seek care or not and from where to seek care. This finding is similar in other patriarchal settings [21].

Care-seeking for maternal and newborn illnesses was influenced by differences in illness recognition and perceived cause of the illness. Home-based care, including home remedies such as baths, herbal tonics, and prayers, were more often described as the first step of care for newborns. This is a similar finding to other studies done in India, Nepal, Bangladesh, and Ethiopia where home-based care is frequently tried first for newborn illnesses [22–24]. For maternal cases, care-seeking was described as more proactive after illness recognition probably as the recognition of severity was easier [15, 16, 20].

Sick mothers and newborns are usually referred from one level to another following Uganda’s pyramidal health care system. However, in 50% maternal death cases and five of the eight cases of newborns deaths, the caretakers were advised to seek care at a higher level while referral with an ambulance to the hospital could not be organized. This finding is similar to other low- and middle-income countries (LMICs) such as Ethiopia where health centers were found to be physical barriers to access [25, 26]. Indeed, in many cases, mothers died on the way during this “empty referral.” These findings call for the need to revisit the role of primary facilities for emergency care. Ideally, primary facility should provide emergency care and prompt referral if needed. However, our interviews indicate that the primary facilities were not able to do so. While upgrading of facilities to provide better emergency care and organize referral is paramount, care-seeking advice during counseling might also talk about signs and symptoms prompting women and families to rather go immediately to a hospital. This could prevent delays. The review of referral and referral transport system was also mentioned as a key priority in the recently published Lancet Maternal Health series [27].

Despite some differences in car-seeking patterns for mothers and newborns, we found that the factors influencing care-seeking were similar for both maternal and newborn and also for the intervention and comparison areas. These ranged from availability of finances for health care, availability of transport, distance to facility, quality of care provided, attitudes of the health workers, and other community support systems. These are similar to findings elsewhere [22, 24, 28]. However, interviews in the intervention area reported that the financial barriers were mitigated through the formation of Women’s savings groups from which funds to seek care could be borrowed. These saving groups were formed as a result of community quality improvement change ideas in the intervention district. In these groups, monthly contributions of 1000 Uganda shillings (about $0.3) are made, and members needing financial support can borrow and pay back later in installments. Members of these groups were therefore reported to borrow money to facilitate care in the event of illness or at the time of delivery, hence a big boost for care-seeking. Our findings support the value of community programs which are gaining popularity for improving care-seeking for maternal and newborn health, especially in LMICs [29–34]. Our qualitative findings also support that CHWs can link the community and formal health facilities by referring patients to facilities for adequate treatment.

One strength of our study is that it provides insight to perceptions and experiences around illness recognition and care-seeking for maternal and newborn illnesses and deaths. This could inform health delivery improvement within the country. Our main limitation is that while our qualitative interviews covered an intervention and comparison area where a community approach was implemented, our study design prevents us from making comparative conclusions. Our sample was small. Another limitation is the possibility of recall bias of the events that could have occurred. We mitigated this by conducting group interviews with the people who were available when the event occurred. Finally, our study setting was rural with few peri-urban communities, and hence, it may not be possible to generalize these findings to purely urban locations.

## Conclusions

Our qualitative findings propose that CHWs and community-based interventions may play a role in early illness recognition and care-seeking for sick mothers and babies. Interviews suggested that recognition of newborn illness was difficult, and care-seeking often limited or delayed. Maternal danger signs were more consistently reported and recognized. The role of primary facilities in providing care for maternal and newborn emergencies needs to be reviewed to prevent delays by following the pyramidal health system.

## Abbreviation

CHW: Community health worker
EQUIP: Expanded Quality Management Using Information Power
FGDs: Focus group discussions
LMICs: Low- and middle-income countries
QI: Quality improvement
QIT: Quality improvement team
UNEST: Uganda Newborn Study
